# Prevalence of Trachoma in Unity State, South Sudan: Results from a Large-Scale Population-Based Survey and Potential Implications for Further Surveys

**DOI:** 10.1371/journal.pntd.0001585

**Published:** 2012-04-10

**Authors:** Tansy Edwards, Jennifer Smith, Hugh J. W. Sturrock, Lucia W. Kur, Anthony Sabasio, Timothy P. Finn, Mounir Lado, Danny Haddad, Jan H. Kolaczinski

**Affiliations:** 1 Medical Research Council Tropical Epidemiology Group, Department of Epidemiology and Population Health, London School of Hygiene and Tropical Medicine, London, United Kingdom; 2 Department of Infectious and Tropical Diseases, London School of Hygiene and Tropical Medicine, London, United Kingdom; 3 Ministry of Health, Juba, Republic of South Sudan; 4 Malaria Consortium South Sudan, Juba, Republic of South Sudan; 5 International Trachoma Initiative, Decatur, Georgia, United States of America; 6 Malaria Consortium Africa Regional Office, Kampala, Uganda; University of Cambridge, United Kingdom

## Abstract

**Background:**

Large parts of South Sudan are thought to be trachoma-endemic but baseline data are limited. This study aimed to estimate prevalence for planning trachoma interventions in Unity State, to identify risk factors and to investigate the effect of different sampling approaches on study conclusions.

**Methods and Findings:**

The survey area was defined as one domain of eight counties in Unity State. Across the area, 40 clusters (villages) were randomly selected proportional to the county population size in a population-based prevalence survey. The simplified grading scheme was used to classify clinical signs of trachoma. The unadjusted prevalence of trachoma inflammation-follicular (TF) in children aged 1–9 years was 70.5% (95% CI: 68.6–72.3). After adjusting for age, sex, county and clustering of cases at household and village level the prevalence was 71.0% (95% CI: 69.9–72.1). The prevalence of trachomatous trichiasis (TT) in adults was 15.1% (95% CI: 13.4–17.0) and 13.5% (95% CI: 12.0–15.1) before and after adjustment, respectively. We estimate that 700,000 people (the entire population of Unity State) require antibiotic treatment and approximately 54,178 people require TT surgery. Risk factor analyses confirmed child-level associations with TF and highlighted that older adults living in poverty are at higher risk of TT. Conditional simulations, testing the alternatives of sampling 20 or 60 villages over the same area, indicated that sampling of only 20 villages would have provided an acceptable level of precision for state-level prevalence estimation to inform intervention decisions in this hyperendemic setting.

**Conclusion:**

Trachoma poses an enormous burden on the population of Unity State. Comprehensive control is urgently required to avoid preventable blindness and should be initiated across the state now. In other parts of South Sudan suspected to be highly trachoma endemic, counties should be combined into larger survey areas to generate the baseline data required to initiate interventions.

## Author Summary

Large parts of South Sudan are thought to be trachoma endemic but baseline data, required to initiate interventions, are few. District-by-district surveys, currently recommended by the World Health Organization (WHO), are often not financially or logistically viable. We therefore adapted existing WHO guidelines and combined eight counties (equivalent to districts) of Unity State into one survey area, randomly sampling 40 villages using a population-based survey design. This decision was based on a trachoma risk map and a trachoma rapid assessment, both identifying the state as likely to be highly endemic. The survey confirmed trachoma as being hyperendemic throughout Unity State, meaning that large-scale intervention should be initiated now. Simulation studies were conducted to determine the likely outcome if fewer (n = 20) or more (n = 60) villages had been sampled, confirming that precision decreased or increased, respectively. Importantly, simulation results also showed that all three sample sizes would have led to the same conclusion, namely the need for large-scale intervention. This finding suggests that district-by-district surveys may not be required for areas where trachoma is suspected to be highly prevalent but that are lacking baseline data; instead districts may be combined into a larger survey area.

## Introduction

Ocular infection with the bacterium *Chlamydia trachomatis* causes trachoma, a disease characterized by inflammation of the conjunctiva, most commonly in children, that can lead to scarring, opacity of the cornea and blindness in later life [1]. Trachoma is responsible for an estimated 3.6% of all cases of blindness, making it the major cause of infectious, preventable blindness worldwide [2]. The disease is largely found in poor, rural communities in low-income countries, where access to water, sanitation and health care is inadequate [3], [4]. Fifty-seven countries, mostly in Africa, are currently known to be trachoma endemic [5]. Globally an estimated 40.6 million people are living with active trachoma (trachoma inflammation-follicular (TF) and/or trachoma inflammation-intense (TI)) and 8.2 million are affected by trachomatous trichiasis (TT) [6], [7]. These recently revised figures differ from 2003 estimates of 84 million cases of active trachoma and 7.6 million cases of TT [6], with the discrepancy being at least partially explained by the availability of better data. The reduction in active disease, however, is also thought to reflect socio-economic development in some of the affected countries and the effective implementation of a comprehensive control strategy [1], [5], [7]. This so-called ‘SAFE’ strategy is being promoted by the Alliance for the Global Elimination of Blinding Trachoma by the year 2020 (GET 2020), established in 1997 by the World Health Organization (WHO). SAFE stands for **S**urgical correction of trichiasis, **A**ntibiotic treatment, **F**acial cleanliness and **E**nvironmental improvement [8], [9]. The WHO recommended thresholds to initiate trachoma control through “A” or “S” are a prevalence of TF in children age 1–9 years of ≥10%, or TT prevalence of ≥1% in people age 15 years or above [1], [10], estimated at a population administrative level for health care delivery or corresponding to a population size of around 100,000.

Apart from providing more up-to-date estimates of the number of trachoma cases, recent reviews also show that a handful of countries carry most of the burden [1], [5], [7]. The newly independent Republic of South Sudan is one of the countries worst affected by both active trachoma and trichiasis, and has been highlighted as being in particularly urgent need of large-scale SAFE intervention [11]. Scaling up of trachoma control in South Sudan has, however, been hampered by a limited understanding of the geographical distribution of the disease, limited baseline data from suspected endemic areas and, ultimately, a shortage of funds to conduct surveys and implement control [12]. The majority of control activities have been undertaken east of the Nile, while a number of prevalence surveys [13], [14] and a recently developed risk map [15] indicate that some areas west of the Nile are in equal need of intervention. Trachoma rapid assessments (TRA) were therefore conducted during 2009 to establish whether there was evidence of ongoing trachoma transmission in two of the suspected trachoma endemic states west of the Nile; Northern Bahr-el-Ghazal and Unity State. The latter was identified as being trachoma endemic throughout, with more than 10% of children aged 1–9 years in all of the thirteen surveyed villages showing signs of TF [16].

Prior to scaling up trachoma interventions in Unity, further survey work was required to generate estimates of trachoma prevalence for planning and monitoring purposes as the TRA methodology was not developed to generate these data [17], [18]. With assistance from the United States Agency for International Development Neglected Tropical Disease (NTD) Control Program, a population-based prevalence survey (PBPS) was therefore conducted across Unity State to generate the required baseline data prior to intervention and investigate risk factors for trachoma and trichiasis in this region. Whilst risk factors for TF in children have been investigated in detail, few studies have specifically looked at risk factors for TT.

Survey work in South Sudan is extremely strenuous, due to the lack of infrastructure and the harsh climate, and financial resources for trachoma-related activities are scarce. To minimize effort and cost it was decided to combine the eight (of nine) counties of Unity State for which there were no baseline data under one survey area. The implementation of a prevalence survey at an administrative level larger than a district or county may reduce the cost and time to conduct baseline prevalence surveys, which can enable national trachoma programs to raise funds and implement control measures sooner. The option of modifying the current approach of district-by-district PBPS surveys to target trachoma interventions has been recognised by other investigators (e.g. [19]) and some alternative study designs are currently under investigation. Here we sampled fewer sites than if county-by-county surveys had been conducted and used computerized simulations to compare the likely results from varying the number of sites surveyed, and hence the overall sample size, in this PBPS surveys with the aim of informing future sampling strategies in areas suspected to be highly trachoma endemic.

## Methods

### Ethical considerations

The protocol for the national trachoma prevalence survey, including the procedure for informed consent as outlined below, had received ethical approval from the Directorate of Research, Planning and Health System Development, Ministry of Health, Government of South Sudan (MoH-GoSS), in 2008. Clearance to conduct the surveys was obtained from the State Ministry of Health, followed by County Health Departments. The study was explained to each member of the selected households and the household head was asked to provide written consent for the entire household to participate in the study. To all illiterate heads of households the consent form was read out, and s/he was requested to consent by providing a thumbprint on the consent form. Due to the large number of individuals that were surveyed, it was considered impractical to obtain written consent from each study participant, and the MoH-GoSS approved this procedure. To document verbal consent, the name of each individual who provided verbal consent was recorded, along with the results from the eye examination. Each inhabitant of the household was requested to provide verbal consent; individuals who did not consent were not examined. Personal identifiers were removed from the dataset before analysis.

### Study site and population

**Figure 1:**
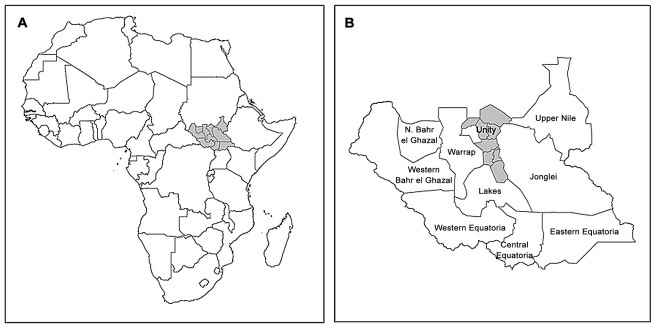
Location of survey area. A) Map of Africa showing location of South Sudan (grey shaded); B) Map of South Sudan showing location of the eight counties surveyed in Unity State (grey shaded).

During March 2010, a PBPS was conducted across Unity State in the north of South Sudan (Figure 1). In 2008, the state population had been estimated at 585,802 people (2008 Census Data), while recent program data from delivery of long-lasting insecticidal nets (LLINs) and mass drug administration (MDA) indicate that the actual population is around 700,000 (Malaria Consortium, unpublished) The inhabitants belong to two ethnic groups, the Nuer and the Dinka, both of which are pastoralists and rely on cattle products for most aspects of their daily lives.

The PBPS followed the standard MoH-GoSS protocol for a national trachoma prevalence survey [20], except for two notable exceptions: 1) The area surveyed here was nearly the entire state, while previous PBPS only surveyed counties or payams [13], [14], [21]–[23]. In South Sudan, the state is the first administrative level, while the county and payam comprise the second and third administrative level, respectively. 2) Based on financial and logistical considerations, it was decided to survey a total of 40 sites across the state, whereas the national protocol suggests that 20 sites should be surveyed per county. The rationale for using a larger study area and population in the present PBPS was that Unity State had already been indicated as highly trachoma endemic [14], [15], [16]. Survey modification as described above was therefore agreed among partners in South Sudan and with the International Trachoma Initiative to ensure that baseline data to target SAFE interventions could be generated quickly and efficiently without compromising its quality.

Unity State is composed of nine counties: Mayom, Rubkona, Pariang, Leer, Guit, Koch, Abiemnhom, Mayendit and Panyijar. Mankien payam in Mayom was surveyed for trachoma in May 2005 [14] and, as no intervention had occurred in the county, Mayom was excluded from the present survey. The total number of villages to be surveyed in each county was determined based on the population size of the county, using population figures from the 2008 census, with the number of village to be surveyed in each county being proportional to its population size. For each county a list of its payams was drawn up to select survey villages. For counties where the number of villages to be surveyed exceeded the total number of payams, one village in each payam was randomly selected and any additional villages were randomly selected from payams with more than ten villages in them. For counties with fewer villages to be surveyed than the total number of payams, payams and the villages to be surveyed within these were randomly selected. Within each village, 20 households were randomly selected, apart from Pariang County where 21 households were sampled in two of the seven study villages. Households were randomly selected using the sketch map and segmentation method [24]. All residents of the household were enumerated, and all those present who gave informed consent were examined.

### Trachoma grading

Ophthalmic Clinical Officers and General Clinical Officers working for the State Ministry of Health in Unity were trained by an experienced ophthalmologist from Uganda on the use of the WHO simplified grading system over the course of three days; two additional ophthalmologists from Uganda attended the training, as they were designated to lead the survey teams. The WHO grading system categorizes trachoma infection according to five grades: TF, TI, trachomatous scarring (TS), TT and corneal opacity (CO) [25]. Two stages of assessment were used to select the best trainees. In the first stage, trainee examiners identified trachoma grades using the WHO set of trachoma slides. Those examiners who achieved at least 80% agreement then proceeded to the second stage of field evaluation. During field evaluation, a reliability study comprising 50 persons of varying age and sex were selected by the ophthalmologist to represent all trachoma grades. Each trainee examiner evaluated all 50 participants independently and recorded their findings on a pre-printed form. Inter-observer agreement was then calculated for each trainee using the ophthalmologists' observation as the “gold standard.” Unfortunately none of the trainees reached at least 80% inter-observer agreement in the field evaluation and it was therefore decided that grading was to be conducted by the two experienced ophthalmologists only, with the trained graders providing support during the survey.

All inhabitants of selected households who provided verbal consent were examined using a torch and a 2× magnifying binocular loupe. Each eye was first examined for in-turned lashes (TT), and the cornea was then inspected for CO. The upper conjunctiva was subsequently examined for inflammation (TF and TI) and scarring (TS). Signs had to be clearly visible in accordance with the WHO simplified grading system in order to be considered present. Trachoma signs only had to be present in one eye for the person to be categorized as suffering from a particular grade of trachoma. Alcohol-soaked cotton swabs were used to clean the examiner's fingers between examinations. Individuals with signs of active trachoma or bacterial conjunctivitis were treated with 1% tetracycline eye ointment and provided with information on face washing and good hygiene practices. Patients with TT or other significant eye conditions were referred to the nearest facility where free surgery was available (i.e. Bentiu Hospital).

### Data management

The data were verified at the end of each survey day and entered on a portable notebook computer using Microsoft Office Excel (Microsoft Corporation, Seattle, USA). The computer was charged during the day by means of a small generator or through mains electricity, where available. Second data entry was conducted by different staff at the Malaria Consortium office after completion of the survey. Range and consistency checks were conducted for all variables in STATA 11.1 software (Stata Corporation, College Station, TX, U.S.A.).

### Statistical analysis

Data were analysed in STATA 11.1. Individuals with missing data for sex and/or age were excluded from the analysis. Overall and for each county, household attribute data were summarised as the mean (standard deviation SD) of the village means of the proportion of households with a particular attribute. The age and sex distribution of the enumerated population was tabulated.

Unadjusted prevalence estimates for trachoma signs are presented with corresponding 95% exact binomial confidence intervals (CI). Adjusted prevalence estimates and corresponding 95% CIs were obtained from random effects regression models for TF, TI and TS in children aged 1–9 years, for TF and TS in children aged 10–14 years and for TS, TT and CO in adults (defined as those aged 15 and above). All models adjusted for age, sex and clustering of cases as between-village variation and between-household variation parameters. Models also adjusted for county to account for underlying unmeasured variation.

Risk factors for TF in children aged 1–9 and for TT in adults (15 years and above) were investigated using random effects logistic regression. Clustering of individuals with signs of trachoma at household and village levels was investigated using random effects regression models, comparing models with and without between cluster variance parameters using the likelihood ratio test (LRT). Where a LRT p-value was <0.1, the model with the additional random effect parameter was retained to obtain adjusted prevalence estimates of signs of trachoma. A stepwise approach was used to build multivariate models in both risk factor analyses, adjusting for age and sex *a priori*. To account for unmeasured covariates and underlying variation within counties, the final multivariate model for TF also adjusted for county. Model fit was assessed by the LRT where inclusion and exclusion criteria of LRT p≤0.1 were applied.

### Estimating trichiasis surgery needs

To estimate the number of individuals requiring TT surgery, population figures for Unity reported by the 2008 census were adjusted upwards by 20%, based on field experience with LLIN distribution and MDA in the area indicating that census figures provided an underestimate of the actual resident population. The number of individuals aged below and above 15 years was calculated for each county (data not shown) using the relative proportions of people in these age categories as established during a detailed population census conducted as part of a recent MDA round in Mayom county (Malaria Consortium, unpublished). The number of individuals in each of the two age categories was then multiplied by the TT prevalence for each county. To arrive at estimates for Mayom county, TT prevalence figures provided by Ngondi *et al.* [14] were used.

### Comparing survey designs

A computerized simulation approach was used to compare the results from the actual survey, which was conducted in forty villages, to the alternatives of sampling twenty or sixty villages. The latter was the maximum number of sites that was deemed feasible to sample (and actual experience showed that sampling of forty sites was already very challenging), while sampling of twenty sites would have resulted from applying the current MoH-GoSS survey protocol to the next administrative level (i.e. the state instead of the county) and would have helped to keep costs to a minimum.

To compare survey designs, a ‘gold standard dataset’ was generated for the United Nations Office for the Coordination of Humanitarian Affairs (UNOCHA) village database, containing information on 823 geolocated settlements in Unity State (excluding Mayom county). In order to generate as realistic ‘gold standard’ data as possible, the spatial characteristics of TF prevalence were assessed by constructing a semi-variogram, which plots the semi-variance – a measure of expected dissimilarity between a given pair of observations – as a function of the distance separating those observation. Prevalence data for active trachoma (TF and/or TI) in children aged 1–9 years were used from this study, as well as from other population-based prevalence surveys conducted in South Sudan, were used in the semi-variogram analysis. All surveys used the WHO simplified grading system and were conducted between 2001 and 2010. This resulted in data from a total of 179 communities in South Sudan, 40 of which were located within Unity State. A logistic transformation was used on prevalence data from these surveys to reduce skew before analysis, and a small constant of 0.01 was added to the raw data to avoid transforming zero values. Empirical semi-variograms were then estimated using these transformed prevalence estimates.

Prevalence data for all 823 settlements in Unity state were then generated using conditional simulation, which uses the semi-variogram parameters to generate multiple possible sets of data (realisations) that maintain the spatial variance characteristics of the source data [26]. One thousand realisations of prevalence data were generated, and back transformed, and those with the median, minimum and maximum overall prevalence were selected. These three realisations allowed an exploration of the performance of survey designs accounting for uncertainty in the simulated dataset. Further information on the use of semi-variograms and conditional simulation in NTD surveys is provided elsewhere [27].

Sampling simulations followed the original PBPS sampling design described in the Unity State survey, selecting a given number of clusters (proportional to population size) randomly from each of the included eight countries. Using the simulated ‘gold standard’ data, the precision of the original sampling plan of forty clusters was compared to twenty and sixty clusters. Sampling simulations were repeated 1,000 times on each realisation and the range of prevalence estimates resulting from surveying twenty, forty or sixty clusters was recorded. Semi-variogram analyses, conditional simulation and sampling simulations were all carried out using bespoke scripts in R 2.10.1 [28].

## Results

### Study population

Overall, households were of a median size of seven inhabitants, but throughout the study area some households were more than twice this size (Table 1). Data summarised across all villages suggest that few heads of households had received formal education or owned a radio (overall, approximately 12% and 21% respectively), but that three quarters of households owned cattle. Flies were commonly observed within and around living areas or on the eyes of children (95% of households). Latrine access was poor (8%) and around half of the respondents reported a journey of more than 15 minutes walk to reach water.

**Table 1:** Study Population.

		Overall	County							
			Abiemn-hom	Guit	Koch	Leer	Mayendit	Panyihar	Pariang	Rubkona
Villages sampled^a^		40	1	3	6	5	5	4	7	9
Median household size (range)		7 (1–26)	7 (2–13)	8 (3–18)	7 (1–26)	7 (2–18)	6 (2–19)	6 (1–16)	6 (2–17)	7 (2–21)
Household attribute^b^, mean (SD)										
	Protected water source^c^	61.1 (43.6)	100 (-)	0	53.3 (51.6)	60.0 (44.2)	92.0 (7.6)	98.8 (2.5)	61.4 (49.1)	48.9 (40.9)
	Water within 15 minute walk	48.3 (38.1)	100 (-)	60.0 (36.1)	59.2 (47.2)	52.0 (27.1)	46.0 (32.7)	33.8 (44.2)	37.9 (45.8)	45.0 (38.0)
	Latrine access	7.5 (17.2)	25.0 (-)	0	8.3 (20.4)	0	3.0 (4.5)	0	2.1 (3.9)	21.7 (27.8)
	Waste disposed >20 metres away	10.5 (15.2)	30.0 (-)	3.3 (2.9)	5.8 (7.4)	0	5.0 (6.1)	3.75 (2.5)	28.4 (26.2)	11.7 (8.7)
	Cattle ownership	76.0 (30.2)	10.0 (-)	93.3 (7.6)	89.2 (12.8)	92.0 (8.4)	87.0 (8.4)	78.8 (25.0)	77.3 (24.5)	51.7 (43.4)
	Flies around the living area^d^	95.0 (8.0)	85.0 (-)	96.7 (5.8)	98.3 (4.1)	94.0 (6.5)	98.0 (2.7)	98.8 (2.5)	97.2 (2.6)	88.9 (13.4)
	Radio ownership	22.1 (20.6)	55.0 (-)	10.0 (5.0)	19.2 (17.2)	21.0 (8.2)	11.0 (2.2)	13.8 (18.0)	13.5 (14.6)	41.7 (27.4)
	No formal education of household head	88.5 (12.7)	60.0 (-)	93.3 (2.9)	95.0 (6.3)	88.0 (9.1)	96.0 (5.5)	92.5 (5.0)	84.3 (15.9)	83.3 (16.6)
**Individuals enumerated** e		**5727**	**145**	**506**	**850**	**677**	**643**	**493**	**965**	**1448**
*Male*		*2437*	*58*	*223*	*372*	*280*	*276*	*180*	*405*	*637*
	<1 year, n (%)	96 (3.9)	0 (0)	8 (1.6)	9 (1.1)	11 (1.6)	11 (1.7)	11 (2.2)	15 (3.7)	31 (2.1)
	1–9 years, n (%)	1268 (52.0)	29 (20.0)	123 (24.3)	209 (24.5)	159 (23.5)	168 (26.1)	107 (21.7)	195 (48.1)	278 (19.1)
	10–14 years, n (%)	257 (10.5)	9 (6.2)	24 (4.7)	44 (5.2)	33 (4.9)	24 (3.7)	12 (2.4)	48 (11.9)	63 (4.3)
	≥15, n (%)	810 (33.2)	20 (13.8)	68 (13.4)	110 (12.9)	77 (11.4)	73 (11.4)	50 (10.1)	147 (36.3)	265 (18.2)
*Female*		*3299*	*87*	*283*	*478*	*397*	*367*	*313*	*560*	*811*
	<1 year, n (%)	85 (2.6)	2 (1.4)	8 (1.6)	6 (0.7)	11 (1.6)	12 (1.9)	8 (1.6)	11 (2.7)	27 (1.9)
	1–9 years, n (%)	1403 (42.5)	29 (20.0)	138 (27.3)	229 (26.9)	181 (26.7)	166 (25.8)	147 (29.8)	216 (53.3)	297 (20.4)
	10–14 years, n (%)	308 (9.3)	15 (10.3)	26 (5.1)	43 (5.0)	41 (6.1)	33 (5.1)	24 (4.9)	38 (9.4)	88 (6.0)
	≥15, n (%)	1500 (45.5)	41 (28.3)	111 (21.9)	200 (23.5)	164 (24.2)	156 (24.3)	134 (27.2)	295 (72.8)	399 (27.4)
**Examination coverage: Enumerated Individuals with examination data, n (% of enumerated individuals)**										
Trachoma signs (1–9 years)	Male	1146 (90.4)	27 (93.1)	111 (90.2)	189 (90.4)	142 (89.3)	150 (89.3)	96 (89.7)	174 (89.2)	257 (92.4)
	Female	1260 (89.8)	27 (93.1)	129 (93.5)	213 (93.0)	159 (87.8)	150 (90.4)	132 (89.8)	190 (88.0)	260 (87.5)
Trachomatous Scarring (all ages)	Male	1783 (73.2)	44 (75.9)	168 (75.3)	285 (76.6)	206 (73.6)	211 (76.4)	135 (75.0)	292 (72.1)	442 (68.7)
	Female	2824 (85.6)	76 (87.4)	248 (87.6)	427 (89.3)	325 (81.9)	323 (88.0)	281 (89.8)	469 (83.8)	675 (82.9)
Trichiasis and Corneal opacity (≥15 years)	Male	362 (44.7)	10 (50.0)	31 (45.6)	50 (45.5)	34 (44.2)	33 (45.2)	21 (42.0)	64 (43.5)	119 (41.8)
	Female	1240 (82.7)	34 (82.9)	94 (84.7)	172 (86.0)	127 (77.4)	141 (90.4)	121 (90.3)	237 (80.3)	314 (78.7)

a Twenty households sampled per village (except Pariang where 21 households were sampled in two villages).

b Proportion of households per village with each attribute calculated and the mean (SD) of village proportions obtained as the county level summary measure.

c Protected source: handpump or well; unprotected: river, stream, pond or swamp.

d Mean (SD) of village proportions of households observed to have more than five flies in or around living areas or on children's faces, excluding around cattle.

e Six missing values for age in males, three missing values for age in females, four missing values for sex; these 13 individuals were excluded from all analyses.

A total of 5,727 individuals were enumerated after exclusion of 13 individuals with missing values for age (n = 9) or sex (n = 4) (Table 1). The distribution of age by sex within enumerated individuals suggested that the census included similar proportions of male and female children aged 1–9, and more females than males aged 15 years and above (Table 1). Ninety percent of enumerated children aged 1–9 years were examined for signs of trachoma. Of the individuals aged 15 years and above more than 80% of women were examined, but only around 45% of male residents were examined (Table 1).

### Prevalence of trachoma signs

The overall adjusted prevalence of TF in children aged 1–9 years was 71% (95% CI: 70–72%). Unadjusted and adjusted prevalence and corresponding 95% CIs were the same as integer values (Table 2). Incorporating children with TI, the prevalence of active trachoma in children aged 1–9 was 84% (adjusted 95% CI: 83–85%). The prevalence of trachomatous scarring in this age group was at most 2%. Amongst 10 to 14 year olds, TF was also highly prevalent (approximately 42%), while scarring was observed in 8% and TT in up to 6% (Table 2).

**Table 2:** Prevalence of Trachoma Signs.

Trachoma Clinical Sign	Examined	Number with sign	Unadjusted Prevalence^a^ (95% CI)	Adjusted Prevalence^b^ (95% CI)
Children aged 1–9 years:				
TF	2406	1696	70.5 (68.6–72.3)	71.0 (69.9–72.1)
TI	2405	561	23.3 (21.6–25.1)	23.0 (22.3–23.6)
AT	2406	2008	83.5 (81.9–84.9)	84.0 (83.3–84.7)
TS	2406	40	1.7 (1.2–2.3)	-
TT	2406	17	0.7 (0.4–1.1)	-
Children aged 10–14 years:				
TF	428	179	41.8 (37.1–46.7)	41.6 (38.3–45.0)
TS	428	35	8.2 (5.8–11.2)	7.5 (5.1–9.9)
TT	428	17	4.0 (2.3–6.3)	-
Adults aged 15 years and above:				
TS	1602	382	23.9 (21.8–26.0)	23.7 (22.5–24.9)
TT	1602	242	15.1 (13.4–17.0)	13.5 (12.0–15.1)
CO	1602	122	7.6 (6.4–9.0)	6.7 (5.3–8.2)

Data are presented on percentage scale.

TF = trachomatous inflammation - follicular, TI = trachomatous inflammation, AT = active trachoma (TF and, or TI), TS = trachomatous scarring, TT = trachomatous trichiasis, CO = corneal opacity.

a exact binomial confidence interval.

b adjusted for age, sex, county, between-village variation and between-household variation using random effects regression models; adjusted estimates not obtained where prevalence very low (≤4%).

Adjusted prevalence result suggest between 12% and 15% of those aged 15 years and above had TT, far exceeding the treatment threshold of 1%, and between 5% and 8% were experiencing CO in one or both eyes (Table 2).

For estimation of all prevalence figures, adjusted values were very similar to unadjusted values even in the presence of between-cluster variation at household and village levels.

### Risk factors for TF in children aged 1–9 years

Independently more TF was seen in children aged 3–5 years, compared to those aged 1–2 years, for children with ocular and, or nasal discharge and for children from households with flies in and around the living areas or on the faces of children (Table 3), after accounting for clustering of TF within households, within villages and adjusting for county. All of these factors remained statistically significant risk factors for TF in children aged 1–9 years in multivariate analyses, which adjusted for these factors, in addition to age and sex *a priori* and clustering as described.

**Table 3:** Risk factors for Follicular trachoma (TF) in children aged 1–9 years.

Characteristic		N	TF, n (%)	Univariate^a^ OR(95% CI)	p-value^b^	Adjusted^c^ OR(95% CI)	p-value^b^
Child level:							
	1–2	570	388 (68.1)	1	<0.001	1	
	3–5	992	755 (76.1)	1.58 (1.23–2.03)		1.61 (1.25–2.07)	<0.001
	6–9	847	555 (65.5)	0.91 (0.71–1.16)		0.99 (0.77–1.28)	
Sex	Male	1146	808 (70.5)	1	0.777	1	
	Female	1260	888 (70.5)	0.97 (0.80–1.18)		1.05 (0.86–1.28)	0.605
Ocular Discharge	No	1451	983 (67.8)	1	<0.001	1	
	Yes	953	713 (74.8)	1.45 (1.18–1.79)		1.25 (1.00–1.58)	0.063
Nasal Discharge	No	1528	1027 (67.2)	1	<0.001	1	
	Yes	870	662 (76.1)	1.56 (1.26–1.94)		1.33 (1.05–1.69)	0.017
Household level:						-	-
Household size^d^	All residents	-	-	0.99 (0.95–1.02)	0.376	-	-
	Children 1–9	-	-	0.99 (0.94–1.05)	0.806	-	-
Household head education	None	2134	1509 (70.7)	1	0.324	-	-
	≥1 year primary	233	168 (72.1)	1.17 (0.80–1.71)		-	-
	≥1 year secondary	28	14 (50.0)	0.54 (0.20–1.44)		-	-
Protected water source^e^	No	990	702 (70.9)	1	0.654	-	-
	Yes	1419	996 (70.2)	0.93 (0.68–1.27)		-	-
Time to water^f^	<15 minutes	1156	821 (71.0)	1	0.682	-	-
	15–30 minutes	472	330 (69.9)	0.86 (0.62–1.20)		-	-
	>30 minutes	781	547 (70.0)	0.96 (0.69–1.33)		-	-
Latrine access^g^	No	2230	1586 (71.1)	1	0.142	-	-
	Yes	176	110 (62.5)	0.70 (0.44–1.12)		-	-
Waste disposal^g^	<20 m from household	2165	1535 (70.9)	1	0.435	-	-
	≥20 m from household	244	163 (66.8)	0.86 (0.59–1.25)		-	-
Cattle ownership	No	539	356 (66.1)	1		-	-
	Yes	1861	1335 (71.7)	1.28 (0.94–1.74)	0.123	-	-
Flies in/around living areas^g^ ^h^	No	110	64 (58.2)	1	0.014	1	-
	Yes	2299	1634 (71.1)	1.91 (1.15–3.17)		1.79 (1.06–3.03)	0.010
Radio ownership^f^	No	1909	1353 (70.9)	1	0.715	-	-
	Yes	500	345 (69.0)	1.05 (0.79–1.40)		-	-

a Univariate effects adjusted for between-household and between-village variation.

b p-value from likelihood ratio test comparing random effects logistic regression models adjusting for between-household and between-village variation with, and without, characteristic of interest.

c Adjusted for variables included in final multivariable regression model as shown, between-household variation, between-village variation and county.

d Variables modelled as continuous measures.

e Protected source: handpump or well; unprotected: river, stream, pond or swamp.

f Self-reported.

g Observed by fieldworker.

h Observed as flies in and around the living quarters (excluding areas around cattle) and, or faces of children; no if recorded as none or few (1–5); yes if recorded as 5 or more flies.

### Risk factors for TT in those aged 15 years and above

Random effects univariate regression models accounted for clustering at household and village level and suggested increased odds of TT in: i) people aged 30 and above, ii) female adults, iii) people with ocular and, or nasal discharge, and iv) possibly also people who needed to travel further to reach water (Table 4). Decreased odds were seen for people: i) living in larger households, ii) having access to a latrine, iii) residing in households that disposed of waste more than 20 metres away, and iv) radio ownership. After adjustment for age, sex and ocular discharge, nasal discharge was no longer associated with risk of TT (p = 0.206). Following the addition of household size, latrine access and time to water were also no longer significant (p>0.1). A final model for TT included age, sex, ocular discharge, household size and distance to waste disposal (Table 4). Evidence of between-village variation disappeared from the final multivariate model, but household clustering was still apparent.

**Table 4:** Risk factors for trichiasis (TT) in those aged 15 years and above.

Characteristic		N	TT, n (%)	Univariate^a^ OR(95% CI)	p-value^b^	Adjusted^c^ OR(95% CI)	b
Child level:							
Age (years)	15–19	244	9 (3.7)	1	<0.001	1	
	20–29	425	29 (6.8)	1.82 (0.81–4.10)		1.50 (0.66–3.39)	<0.001
	30–39	355	41 (11.6)	3.46 (1.57–7.63)		3.14 (1.43–6.91)	
	40 and above	579	163 (28.2)	12.4 (5.79–26.4)		10.4 (4.89–22.2)	
Sex	Male	362	34 (9.4)	1		1	
	Female	1240	208 (16.8)	2.01 (1.32–3.05)	0.001	2.49 (1.58–3.94)	<0.001
Ocular Discharge	No	1409	175 (12.4)	1	<0.001	1	
	Yes	187	64 (34.2)	3.75 (2.52–5.59)		3.33 (2.15–5.15)	<0.001
Nasal Discharge	No	1567	226 (14.4)	1	0.002	-	
	Yes	24	10 (41.7)	4.82 (1.84–12.6)		-	
Household level:							
Household size^d^	Overall number of inhabitants	-	-	0.92 (0.88–0.97)	0.002	0.95 (0.90–1.00)	0.046
Number of 1–9 year olds in household with TF	0	248	42 (16.9)	1	0.541	-	
	1	375	53 (14.1)	0.80 (0.48–1.36)		-	
	2	368	63 (17.1)	0.98 (0.59–1.64)		-	
	≥3	612	84 (13.7)	0.76 (0.47–1.23)		-	
Child aged 1–14 years with TT in household	No	1537	228 (14.8)	1	0.357	-	
	Yes	66	14 (21.2)	1.43 (0.67–3.04)		-	
Household head education	None	1395	224 (16.1)	1	0.143	-	
	≥1 year primary	164	16 (9.8)	0.61 (0.32–1.16)		-	
	≥1 year secondary	33	2 (6.1)	0.37 (0.07–1.88)		-	
Protected water source e	No	621	98 (15.8)	1	0.829	-	
	Yes	982	144 (14.7)	0.96 (0.63–1.44)		-	
Time to water^f^	<15 minutes	788	95 (12.1)	1	0.076	-	
	15–30 minutes	257	51 (19.8)	1.69 (1.05–2.73)		-	
	>30 minutes	558	96 (17.2)	1.45 (0.95–2.20)		-	
Latrine access^g^	No	1467	233 (15.9)	1	0.025	-	
	Yes	135	9 (6.7)	0.40 (0.18–0.93)		-	
Waste disposal^g^	<20 m from household	1422	227 (16.0)	1	0.028	1	0.037
	≥20 m from household	181	15 (8.3)	0.49 (0.26–0.95)		0.51 (0.26–0.98)	
Cattle ownership	No	447	52 (11.6)	1	0.193	-	
	Yes	1151	188 (16.3)	1.34 (0.86–1.82)		-	
Flies in/around living areas^g^ ^h^	No	89	13 (14.6)	1	0.828	-	
	Yes	1514	229 (15.1)	0.92 (0.44–1.93)		-	
Radio ownership^f^	No	1198	196 (16.4)	1	0.092	-	
	Yes	405	46 (11.4)	0.70 (0.45–1.07)		-	

a Univariate effects adjusted for between-household and between-village variation.

b p-value from likelihood ratio test comparing random effects logistic regression models adjusting for between-household variation with, and without, characteristic of interest.

c Adjusted for variables included in final multivariable regression model as shown and between-household variation.

d Variables modelled as continuous measures.

e Protected source: handpump or well; unprotected: river, stream, pond or swamp.

f Self-reported.

g Observed by fieldworker.

h Observed as flies in and around the living quarters (excluding areas around cattle) and, or faces of children; no if recorded as none or few (1–5); yes if recorded as 5 or more flies.

### Trichiasis surgery needs

The total population of Unity was estimated to be 702,961 based on recent delivery of interventions by Malaria Consortium. Of these, 47% were assumed to be aged 15 or above. Using TT prevalence figures for each county to calculate surgery needs, this meant that a total of 54,178 individuals (lower and upper bounds = 40,327–71,119) are likely to require TT surgery in Unity State, with the majority (91%) being aged 15 or above.

### Effect of the number of sites surveyed

**Figure 2:**
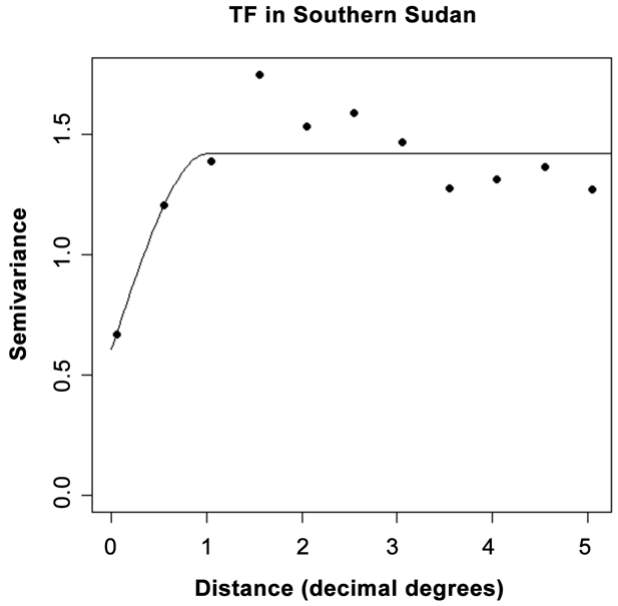
Semi-variogram of trachoma prevalence in 823 communities in eight counties of Unity State, South Sudan. Omnidirectional semi-variogram and best-fit line of exponential spatial models for logistically transformed prevalence data is presented. Parameter values of the fitted spatial model were range = 1.0125, sill = 0.8174, nugget = 0.6064. Note: at the equator, one decimal degree equates to approximately 110 kilometres.

Available community level data from the whole of South Sudan had a median prevalence of 53% (range 0–100%). The semi-variogram generated from these data showed that spatial autocorrelation was present up to approximately one decimal degree (∼110 km) (Figure 2).

**Figure 3:**
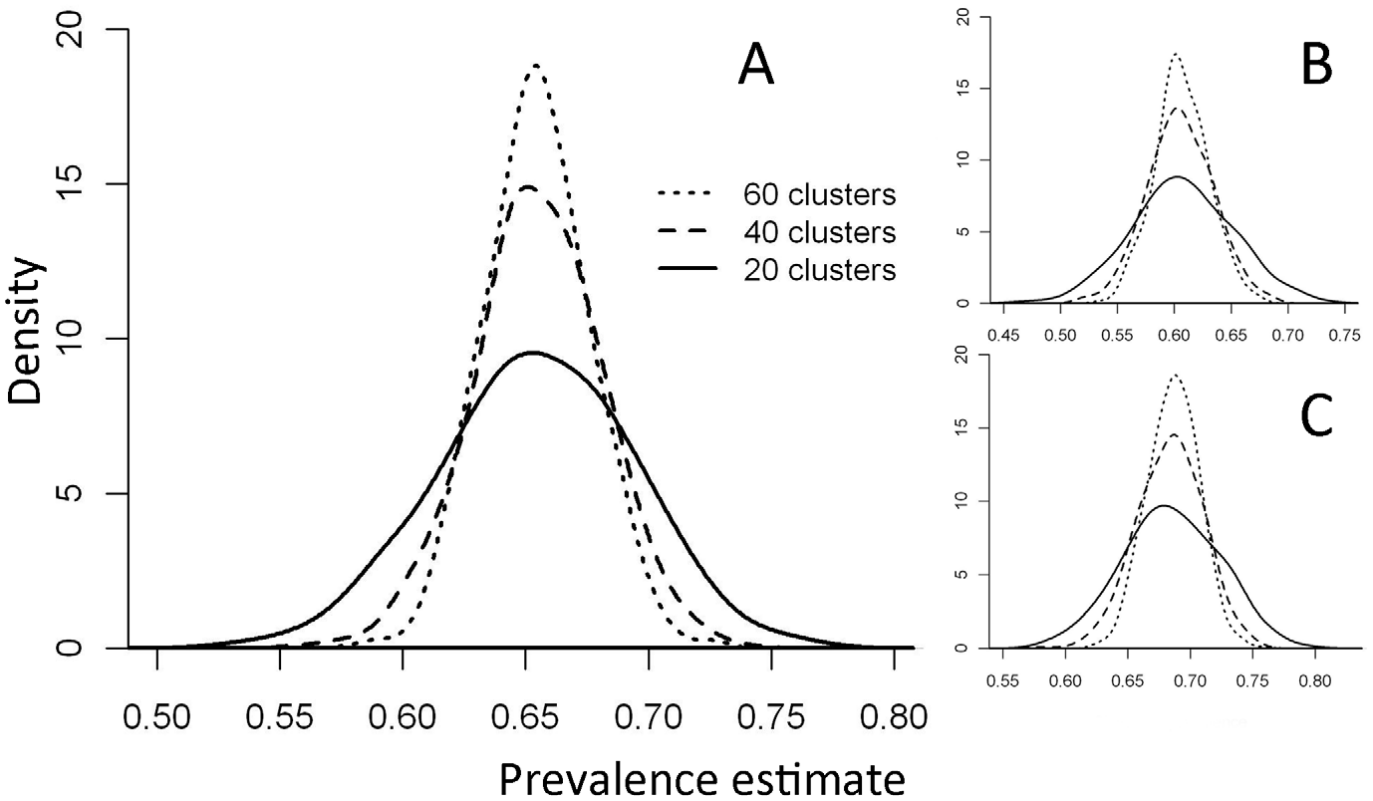
Filled density plots showing the results of the sampling simulations. Simulations were run on realisations with the median (A), minimum (B) and maximum (C) overall prevalence.

The simulated results from each of the alternative sample designs are presented in Table 5, and confirm a reduction in precision associated with reducing the number of clusters by half and an increase in precision when additional clusters are included. Theoretically the median estimate should be the same for all sampling scenarios of a given realisation. In these simulations, the median estimate for each realisation was slightly higher in designs with a higher number of clusters. This is a function of the PBPS sampling strategy, which places relatively more weight on counties with a smaller population size in cases where the calculated number of clusters is rounded up to equal that in different sampling scenarios. In these simulations, Abiemnhom had a low population proportion (0.04) and a lower predicted prevalence. One site was included from this county in two sampling scenarios (40 and 20 clusters), giving this site a slightly greater weight in the smaller sample. If sites had been selected randomly from the entire state (the next highest population level), then the simulated median prevalence would have been the same for all sampling scenarios. The overall effect on the results is negligible, and the confidence intervals presented in Table 5 are the key parameter. Importantly, the lower range of the credible interval remains well over the 40% threshold in all scenarios, indicating that any of these strategies would provide good evidence that this area is hyperendemic for trachoma. This is best illustrated in Figure 3, which compares the three sampling strategies for the median, minimum and maximum realisations using filled density plots.

**Table 5:** Results from sampling simulations in Unity State.

Simulated prevalence and range (95% CI)	Realisation from posterior distribution		
	Median estimate	Minimum estimate	Maximum estimate
Model A : 60 clusters	0.656 (0.610–0.694)	0.604 (0.558–0.651)	0.690 (0.644–0.731)
Model B: 40 clusters	0.655 (0.607–0.712)	0.607 (0.548–0.665)	0.684 (0.632–0.735)
Model C: 20 clusters	0.651 (0.567–0.731)	0.601(0.512–0.682)	0.684 (0.603–0.757)

Sampling simulations were repeated 1000 times, assuming 500 children were enrolled in each cluster (village) and 50 children sampled from each cluster.

## Discussion

Data from a previous survey [14], a national trachoma risk map [15] and TRA [16] had indicated trachoma to be endemic throughout Unity State, but provided an insufficient baseline to initiate interventions. The present study therefore set out to generate the required data. The results established that trachoma prevalence in Unity State far exceeds the WHO recommended threshold of 10% TF prevalence in children aged 1–9 years for initiation of antibiotic MDA [10] and of 1% for TT in adults for surgery provision. Alongside the obvious need for MDA and surgery, both the ‘F’ and ‘E’ components of the SAFE strategy [29] will need to be scaled up to address key trachoma risk factors, hence maximising the impact of intervention and contributing towards sustaining trachoma control once it has been achieved.

The very high prevalence of trachoma found in the eight surveyed counties is consistent with results from previous work in Mayom county of Unity State [14], predictions generated by a national trachoma risk map [15] and findings from most other trachoma surveys in South Sudan [see map in [22]].

The uniformly high prevalence of trachoma found across Unity State is also reflected in the results from the sampling simulation. These provide evidence that more economical survey designs, sampling fewer clusters, can be used for decision making in areas where trachoma is likely to be hyperendemic. This may support a move towards regional level surveys in areas where there is evidence that the prevalence of trachoma is high. Hyperendemic areas, such as Unity State, will have more uniform treatment requirements than areas of lower prevalence, in which individual foci may be above treatment thresholds but overall, the health district does not qualify for MDA. In meso- and hypo-endemic areas, higher resolution surveys will therefore be required to capture high prevalence foci and sufficiently understand spatial variation in disease and treatment needs. Lot Quality Assurance Sampling, which allows decisions on control to be made using small sample sizes, has previously been explored in Malawi [30] and Vietnam [31] and may offer a potential solution. Further exploration of this principle would be possible using data from states to the west of Unity State, where trachoma prevalence is predicted to be much lower [15].

Compared to data from other trachoma endemic areas of Sub-Saharan Africa, the levels of TF and TT found in Unity State were considerably higher. For example, in Kenya's Samburu district, prevalences of 35% TF were reported in children below 10 years and 6% TT in adults above 14 years of age. The same survey covered five other districts and found prevalence of both signs to be lower in all of them [32]. A regional survey in Chad found that TF was present in 31.5% of children under 10 years and that 1.5% of women over 14 years had signs of TT [33]. In Ethiopia, the national prevalence of TF in children below 10 years has been reported as 26.2%, while 3.1% of women above the age of 14 years showed signs of TT [34]. These proportions obviously vary considerably throughout the country; an overall prevalence of 32.7% TF and 6.2% TT has been reported from Amhara regional state [35], Ethiopia's worst affected region [34]. Unity State, like other parts of South Sudan [13], [22], is therefore among the most severely affected by trachoma in Africa.

The present study identified a number of risk factors for trachoma in Unity State. For TF, ocular and/or nasal discharge and the presence of flies in and around the living areas or on children's faces were associated with an increased risk of trachoma infection, and children between 3–5 years of age were at highest risk. Risk factors for TT in those aged 15 years and above were age, sex, ocular discharge, number of children residing in the household and time (as a proxy for distance) to the nearest water collection site. These observations are consistent with our general understanding of trachoma epidemiology [1] and findings of other studies in South Sudan [36], [37] and in the region [38]–[41].

While eye discharge was identified as a TT risk factor in adults we think that it is likely to be a consequence of TT rather than a cause and may also be indicative of being generally unwell, which could be due to having eye health problems. Latrine provision and close access to water were both limited throughout the study villages, but the strong associations with increased risk of TT is suggestive of poorer hygiene in those with more pronounced eye problems, as were poor waste disposal practices. MDA of antibiotics is unlikely to have an effect on these risk factors, highlighting the importance of health education and environmental improvements as part of a comprehensive control programme [39].

Adult females, rather than males, were at much higher risk of having TT, which is generally thought to be due to the close contact of women with children, children being the main reservoir of infection [1]. Fewer adult males were examined from the enumerated population and if these unexamined males were unaffected by the later stages of disease, the sex effect seen would be greater. We found that TT increased with decreased household size and distance to waste disposal. Increased TT in adults in smaller households, after adjustment for other factors, may also be indicative of adults with TT living in isolation and poverty rather than a smaller household being a risk factor for TT.

The present study has a number of limitations. Unlike other PBPS conducted in South Sudan and elsewhere, relatively few villages were sampled in each county, which may have affected the precision of the prevalence estimates for the State, particularly if there was much variation in prevalence within the survey area. Sampling of a total of 40 sites over a large geographical area was, however, considered justified because GIS-based risk-mapping and TRA data had provided a reasonable indication that Unity State was trachoma endemic throughout [15], [16]. As indicated by the summary measures of household attributes there was some variability between counties and this will not have been captured in as much detail as sampling of 20 sites per county would have allowed. It is nevertheless thought that the overall results generated by the study are reflective of the population in the study area and suitable for decision-making on intervention, particularly as WHO thresholds are very clearly exceeded at all population levels. In the course of scaling up the SAFE strategy it may, however, be found that TT surgery requirements need to be adjusted up- or downwards. This is because we were unable to examine 55% of the enumerated adult males, meaning that the prevalence of TT, CO and visual impairment in the study area could differ somewhat from the estimates provided here.

It was not possible to collect DNA samples from children in order to conduct laboratory testing to detect *Chlamydia trachomatis*. It has been suggested that in a high prevalence setting, prior to availability of mass antibiotic treatment, a diagnosis of TF may not reflect infection with *C. trachomatis* in 30% of children aged 1–10 [42]. In Unity State, a reduction of 30% in overall prevalence would still imply that the area is hyper-endemic and in desperate need of MDA. Data used to generate simulated realisations were also based on estimates of TF and/or TI in children aged 1–9 years. The inherent measurement error in using clinical signs as opposed to infection prevalence is also a limitation of the realisations, which were based on real survey data. Additionally, generation of the gold standard database is necessary to evaluate alternative sampling designs, but involves a number of assumptions. Included surveys span nearly a decade and it is possible that the spatial characteristics of infection may have changed over time. However, the lack of MDA in many suspected trachoma endemic areas and the general absence of health infrastructure and services in South Sudan makes it unlikely that the spatial distribution of infection will have changed significantly. Even if there had been changes in the rest of South Sudan over time, sampling simulations were conducted within Unity State and this area will have been most strongly influenced by the recent survey reported here.

Much remains to be done if South Sudan is to eliminate blinding trachoma by 2020. As a first step, a comprehensive intervention program needs to be scaled up across Unity state, now that the baseline data are available. Secondly, there is a need for further large-scale surveys, such as the one reported here, in other states. As much as half of the country may need to be targeted with SAFE interventions [15], but for many areas this still needs to be confirmed. For large suspected endemic areas with little to no available data, such as Upper Nile State and parts of Warrap, Lakes and Central Equatoria States, a two-step procedure such as that used in Unity State would seem the most effective way to get control activities quickly under way. TRAs should be conducted to determine whether there is evidence of transmission in suspected endemic areas. Where the proportion of children with signs of TF is found to be very high, counties could then be combined under one survey area to more quickly identify SAFE intervention needs.

## Supporting Information

Checklist S1 **STROBE checklist.**
(DOC)
